# Prevalence of Pathogens in Poultry Meat: A Meta-Analysis of European Published Surveys

**DOI:** 10.3390/foods7050069

**Published:** 2018-05-03

**Authors:** Andiara Gonçalves-Tenório, Beatriz Nunes Silva, Vânia Rodrigues, Vasco Cadavez, Ursula Gonzales-Barron

**Affiliations:** CIMO Mountain Research Centre, School of Agriculture, Polytechnic Institute of Bragança, Campus de Santa Apolónia, 5301-855 Bragança, Portugal; andiaragoncalves@gmail.com (A.G.-T.); beatriznsilvaa@gmail.com (B.N.S.); vania.rodrigues@ipb.pt (V.R.); vcadavez@ipb.pt (V.C.)

**Keywords:** chicken, *Listeria monocytogenes*, *Campylobacter*, *Salmonella*, *Staphylococcus aureus*

## Abstract

The objective of this study was to investigate and summarize the levels of incidence of *Salmonella* spp., *Listeria monocytogenes*, *Staphylococcus aureus* and *Campylobacter* spp. in poultry meat commercialized in Europe. After systematic review, incidence data and study characteristics were extracted from 78 studies conducted in 21 European countries. Pooled prevalence values from 203 extracted observations were estimated from random-effects meta-analysis models adjusted by pathogen, poultry type, sampling stage, cold preservation type, meat cutting type and packaging status. The results suggest that *S. aureus* is the main pathogen detected in poultry meat (38.5%; 95% CI: 25.4–53.4), followed by *Campylobacter* spp. (33.3%; 95% CI: 22.3–46.4%), while *L. monocytogenes* and *Salmonella* spp. present lower prevalence (19.3%; 95% CI: 14.4–25.3% and 7.10%; 95% CI: 4.60–10.8%, respectively). Despite the differences in prevalence, all pathogens were found in chicken and other poultry meats, at both end-processing step and retail level, in packed and unpacked products and in several meat cutting types. Prevalence data on cold preservation products also revealed that chilling and freezing can reduce the proliferation of pathogens but might not be able to inactivate them. The results of this meta-analysis highlight that further risk management strategies are needed to reduce pathogen incidence in poultry meat throughout the entire food chain across Europe, in particular for *S. aureus* and *Campylobacter* spp.

## 1. Introduction

Among the group of infectious bacteria, *Salmonella* spp., *Listeria monocytogenes*, enterotoxigenic *Staphylococcus aureus* and *Campylobacter* spp. are the main contaminants in food due to their high occurrence worldwide and for being major causes of gastroenteritis in humans [1,2].

The food poisoning symptoms caused by *Salmonella* spp. and *Campylobacter* spp. are usually abdominal cramps, fever, vomiting and diarrhea, which is often bloody in the case of the latter pathogen [3]. Listeriosis is the foodborne disease more likely to lead to hospitalization and mortality, and symptoms can include headache, stiff neck, confusion, loss of balance and convulsions in addition to fever and muscle aches. In pregnant women, listeriosis is particularly dangerous as it can lead to miscarriage, stillbirth, premature delivery, or life-threatening infection of the new-born [3]. *S. aureus* food poisoning symptoms include diarrhea, nausea, stomach cramps and vomiting [3]. Although it is not regarded as a disease as severe as listeriosis, for instance, in some rare cases, acute staphylococcal enterotoxicosis can cause death due to complications [4].

Epidemiological studies have suggested that products of poultry origin are among the most common vehicles for transmission of *Salmonella* spp. and *Campylobacter* spp. [5,6]. According to the European Food Safety Authority (EFSA), there is ample evidence that *Campylobacter* spp. is a foodborne hazard related to poultry meat, by cross-contamination from contaminated broiler meat to ready-to-eat foods [7]. It is estimated that 20% to 30% of human cases of campylobacteriosis are caused by handling, preparation, and consumption of broiler meat, while 50% to 80% may be attributed to the chicken reservoir as a whole [8]. EFSA also points *Salmonella* spp. as a high priority pathogen regarding poultry meat inspection [8], as poultry meat and poultry products are common sources of sporadic and outbreak-related cases of salmonellosis [9].

Listeriosis is usually associated with ready-to-eat products, including products made of poultry meat, in which contamination has occurred before or during processing, followed by growth during prolonged refrigerated storage [8]. With regard to *S. aureus*, it is considered that the risk of disease is more related with improper hygiene and storage throughout the food chain than its occurrence in raw meat, as this bacterium can be found naturally in poultry meats but also in the environment [8], facilitating cross-contamination between surrounding areas and poultry products.

It is important to perform research on pathogens’ detection in food to assess the main zoonotic disease carriers and to combine this information with other available in literature, through meta-analysis. By doing this, it is possible to address a wide range of food safety questions, such as the incidence of diseases and the risks imposed to public health [10]. Hence, the objective of this study was to conduct a systematic review of the incidence of *Salmonella* spp., *L. monocytogenes*, *S. aureus* and *Campylobacter* spp. in raw poultry meat at the end of the processing stage and sold at European retail establishments. In addition, meta-analysis was applied to quantitatively combine and compare the incidence of each pathogen: (i) in poultry meat as a whole; (ii) in chicken vs. other poultry meats; (iii) in poultry by different sampling stages; (iv) in chicken or other poultry meats according to distinct types of cut; (v) in chicken or other poultry meats by packaging status; and (vi) in poultry by cold preservation type.

## 2. Materials and Methods

As a statistical analysis of a large collection of results from published studies, meta-analysis aims to integrate and interpret the findings to achieve comprehensive conclusions that the individual studies alone would not demonstrate clearly [11]. In this study, the *population* is defined as raw poultry meat surveyed at the end of the processing stage or at retail establishments in Europe, while the *measured outcome* is the detection of pathogens. Electronic literature search was carried out in Scopus and Scielo databases to find articles and official reports published since 2000 summarizing the incidence of microbiological hazards in chicken meat produced and commercialized in Europe. The search was done systematically and aimed to find quality studies validated by the scientific community. Grey literature was not procured for two reasons; on the one hand, to avoid data validity concerns; and, on the other hand, to avoid data duplication, since it is probable that high-quality theses and reports be also published in peer-reviewed journals.

The bibliographic searches were undertaken using a formula that combined terms regarding the existence (prevalence, incidence, occurrence, quality, contamination, survey, sampling) of pathogens (*Campylobacter, Listeria monocytogenes, Salmonella, Staphylococcus aureus*) in the selected products (chicken, turkey, broiler, poultry), while excluding other meta-analysis studies, systematic reviews and articles regarding feed and where products were artificially inoculated (artificial, inocul*, spiked). All the terms were combined by properly applying the AND, OR and AND NOT logical connectors.

After assessing all the information from the recovered publications, seventy-eight primary studies [6,12,13,14,15,16,17,18,19,20,21,22,23,24,25,26,27,28,29,30,31,32,33,34,35,36,37,38,39,40,41,42,43,44,45,46,47,48,49,50,51,52,53,54,55,56,57,58,59,60,61,62,63,64,65,66,67,68,69,70,71,72,73,74,75,76,77,78,79,80,81,82,83,84,85,86,87,88] published from 2000 until April 2017 were considered appropriate for inclusion as they satisfied the following criteria: (i) reported outcomes from European processing units or retail establishments; (ii) use of approved microbiological methods; and (iii) presenting sufficient and extractable data (in presence/absence format).

As the incidence of microbial hazards in poultry meat is a binary trait (i.e., a sample tests either positive or negative for the pathogen), the parameter used to measure the effect size was the raw proportion *p* (calculated as the number of successes or positive samples, *s*, divided by the total sample size, *n*). Thus, from each primary study, the number of positive samples *s* and the total number *n* were extracted, and *p* was calculated. Additionally, study characteristics, such as country of origin, survey’s year, sample weight (in g), poultry class (chicken or other poultry), type of meat cut (i.e., whole carcass, pre-cut, minced meat or giblets), sampling stage (i.e., end of processing or retail), packaging status (i.e., unpacked or packed in modified atmosphere packaging—MAP,) and cold preservation type (i.e., chilled or frozen) were annotated.

Several multilevel meta-analysis models were fitted to appropriate data subsets in order to estimate overall or pooled prevalence values for: (i) each pathogen in poultry meat as a whole; (ii) each pathogen in chicken and in other poultry meats; (iii) each pathogen in poultry meat disaggregated by sampling stage; (iv) each pathogen in chicken and in other poultry meats by type of cut; (v) each pathogen in chicken and in other poultry meats by packaging status; and (vi) each pathogen in poultry meat by different cold preservation types. For a detailed explanation on the calculation of study’s weight (precision) and multilevel meta-analysis modelling for incidence data, refer to Xavier et al. [11] and Viechtbauer et al. [89]. Meta-analysis models, and Galbraith and forest plots were built in RStudio version 1.0.136 (RStudio, Boston, MA, USA) using the ‘metafor’ [89] and ‘sqldf’ [90] packages.

## 3. Results

Following study quality checking, a total of 203 observations of positive and negative results of incidence of foodborne pathogens in poultry meats were retrieved.

Most of the observations excerpted from the primary studies were regarding *L. monocytogenes* (*n* = 75), followed by *Salmonella* spp. (*n* = 51) and *Campylobacter* spp. (*n* = 50). *S. aureus* was the pathogen with the fewest observations retrieved (*n* = 27). It is worth mentioning that the meta-analysis results represent a synthesis of 21 European countries; namely, Austria, Bulgaria, Croatia, Denmark, Estonia, Finland, Germany, Greece, Hungary, Ireland, Italy, the Netherlands, Poland, Portugal, Romania, Serbia, Spain, Sweden, Switzerland, UK, and Turkey. Therefore, the pooled prevalence estimates, to be presented as follows, cannot be generalized to other European countries.

### 3.1. Incidence of Pathogens in Poultry Meat

**Figure 1:**
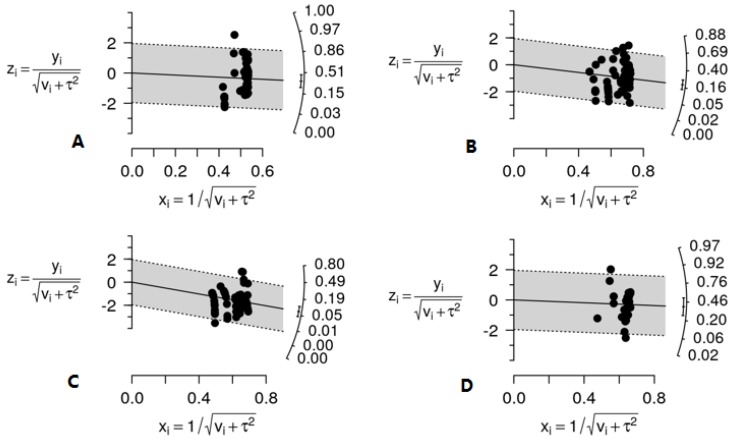
Galbraith plots of the meta-analyses of the incidence of the four pathogens in poultry meat surveyed at the end-processing stage and at retail establishments in Europe; *Campylobacter* spp. overall prevalence 33.3%; 95% CI: 22.3–46.4% (**A**); *L. monocytogenes* overall prevalence 19.3%; 95% CI: 14.4–25.3% (**B**); *Salmonella* spp. overall prevalence 7.10%; 95% CI: 4.60–10.8% (**C**); *S. aureus* overall prevalence 38.5%; 95% CI: 25.4–53.4% (**D**).For each observation i, y_i_ denotes the logit-transformed incidence; v_i_ the sampling variance; τ^2^ the between-study variance; x_i_ the incidence value’s precision; and z_i_, the standardized logit-transformed incidence

The overall prevalence of the four pathogens in poultry meat can be read from the Galbraith plots presented in Figure 1. In a meta-analytical Galbraith plot, the standardized logit transformation of the incidence value (*y*-axis) taken from a study is plotted against its precision (*x*-axis), and the meta-analysis solution is shown on the prevalence scale to the right. None of the plots showed evidence of outliers, yet all of them suggest the presence of heterogeneity among the observations extracted from the literature, fact also corroborated by the intra-class correlation *I*^2^ values, which in most cases were higher than 0.25 (Table 1). In meta-analysis, between-study variability can be considered as significant when it represents at least 25% of the total variability in the outcome measure.

Overall, *S. aureus* bears the highest pooled prevalence in poultry meat (38.5%; 95% CI: 25.4–53.4%), followed by *Campylobacter* spp. (33.3%; 95% CI: 22.3–46.4%) and *L. monocytogenes* (19.3%; 95% CI: 14.4–25.3%), while *Salmonella* spp. was the pathogen of lowest prevalence (7.10%; 95% CI: 4.60–10.8%).

### 3.2. Incidence of Pathogens by Poultry Type: Chicken Vs. Other Poultry Meats

The meta-analysis results on the prevalence of pathogens in poultry meat by poultry class are presented in Table 1. In chicken meat, *Campylobacter* spp. presented the highest prevalence (48.6%; 95% CI: 28.1–69.6%), followed by *S. aureus* (39.9%; 95% CI: 19.8–64.0%). For other poultry meats, such as turkey and duck, the microorganism of highest prevalence was *S. aureus* (43.1%; 95% CI: 20.4–69.2%), followed by *Campylobacter* spp. (23.0%; 95% CI: 9.00–47.2%). Despite the outcomes of this meta-analysis are, in principle, not directly comparable to the official incidence figures from the latest EFSA report [91]—since the former involves a precision-weighted average of results spanning from 2000 to 2017, while the latter compiles non-weighted averages from 2016—the EFSA’s high mean incidence of *Campylobacter* in broiler meat (36.7%) and turkey meat (11.0%) [91] supported our findings. The pooled prevalence estimates for *Salmonella* spp. in chicken meat (3.2%; 95% CI: 1.6–6.4%) and other poultry meats (5.7%; 95% CI: 2.7–11.9%) were lower than the mean incidence values of *Salmonella* spp. in fresh broiler meat (6.4%) and fresh turkey meat (7.7%) from the 2016 EFSA report [91], although such values were still within the confidence intervals of the meta-analytical estimates. The pooled prevalence estimates of *S. aureus* in chicken/other poultry and of *L. monocytogenes* in chicken (21.0%; 95% CI: 14.1–30.1%) and other poultry meats (12.9%; 95% CI: 7.80–20.4%) could not be contrasted against EU official figures since neither *L. monocytogenes* nor *S. aureus* in raw meats or meat preparations are of compulsory notification to EFSA.

### 3.3. Incidence of Pathogens by Type of Cut

For different types of cuts commercialized (pre-cut, whole carcass, giblets, mince), it was observed that, in whole carcasses, *S. aureus* is the pathogen of greatest concern as it has the highest prevalence (72.6%; 95% CI: 38.6–91.8%) while in pre-cut meat, *Campylobacter* spp. was the pathogen of greatest prevalence (30.5%; 95% CI: 11.5–59.5%). Observations regarding giblets and minced meat were only found for two pathogens each (*Salmonella* spp. and *S. aureus* in giblets; and *L. monocytogenes* and *Salmonella* spp. in minced meat). *S. aureus* had the highest prevalence in giblets (43.0%; 95% CI: 16.9–73.6%) while *L. monocytogenes* was the pathogen most frequently recovered in minced meat (19.0%; 95% CI: 7.30–40.9%).

This data partition, by type of cut, allows some insight into the sources of heterogeneity between studies. Overall, for *Salmonella* spp., *S. aureus* and *Campylobacter* spp., the different types of meat cuts were able to explain 15 to 25% of this variability (*R*^2^). However, for *L. monocytogenes*, this moderator, cut type, is seemingly not a source of between-study variability.

### 3.4. Incidence of Pathogens by Sampling Stage

The meta-analysis results on the incidence of pathogens by sampling stage are shown in Table 2. *S. aureus* presented the highest prevalence in poultry meat surveyed at retail level (51.6%; 95% CI: 31.8–70.9%) and at the end-processing stage (38.1%; 95% CI: 14.2–69.7%), followed by *Campylobacter* spp. (44.3%; 95% CI: 28.1–61.8% at retail level; 30.7%; 95% CL: 11.8–59.4% at end-processing). *Salmonella* spp. had the lowest prevalence both at the end-processing stage (5.40%; 95% CI: 1.60–16.1%) and at retail level (10.4%; 95% CI: 5.30–19.3%). Except for *L. monocytogenes*, the recovery of pathogens in poultry meat at retail (i.e., sampled from supermarkets and butcher’s) is more frequent than when sampled in factories at the end of processing (Table 2).

### 3.5. Incidence of Pathogens by Packaging Status

The results of the meta-analysis performed on the incidence of pathogens by packaging status are presented in Table 3. Overall, *Campylobacter* spp. was the pathogen of greatest prevalence in either packed or unpacked poultry meats, with the highest prevalence occurring in chicken (47.2%; 95% CI: 19.4–76.9% in packed chicken; 47.1%; 95% CI: 13.1–84.1% in unpacked chicken). Further, it can be observed that, for the other three bacteria, all unpacked products revealed higher prevalence of pathogens than the packed ones, which can be explained by the fact that packed products have a physical barrier which helps to decrease and slow down the meat deterioration processes [92].

Contrarily to the moderator “type of cut”, this moderator “packaging status” explained only marginally the between-study variability present in the data.

### 3.6. Incidence of Pathogens by Cold Preservation Type

The results of the meta-analysis on the incidence of pathogens by cold preservation type are shown in Table 4. In chilled poultry meat, *S. aureus* was the pathogen of highest prevalence (46.9%; 95% CI: 30.8–63.7%), followed by *Campylobacter* spp. (43.9%; 95% CI: 24.2–65.7%). *Salmonella* spp. shows the lowest prevalence (7.10%; 95% CI: 4.10–12.0%) in chilled meat.

As few data available were available for freezing preservation, conclusions regarding contaminated frozen poultry meat can only be drawn for one pathogen, *Campylobacter* spp. From the prevalence values obtained in chilled and frozen meats, it can be stated that freezing affects the growth and recovery of *Campylobacter* spp., thus ensuring lower prevalence of this pathogen in poultry meats (9.80%; 95% CI: 3.20–26.3%) and improving food safety.

**Figure 2:**
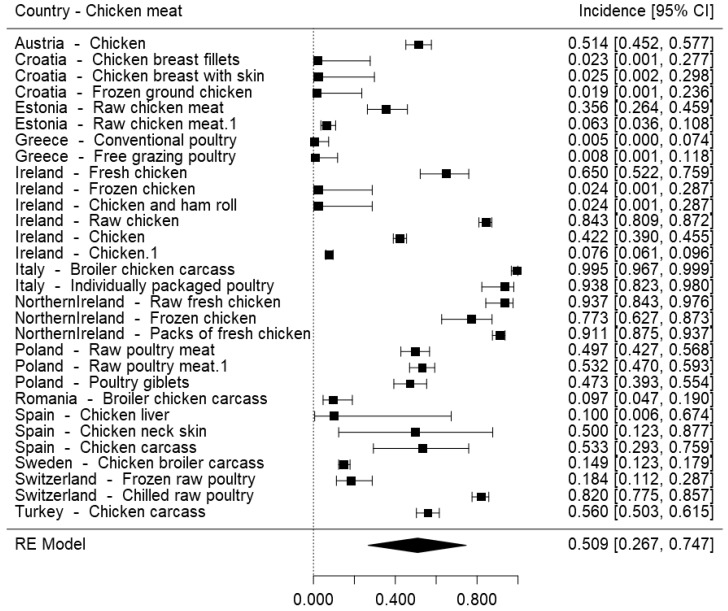
Forest plot of the incidence of *Campylobacter* spp. in chicken meat surveyed in European establishments.

**Figure 3:**
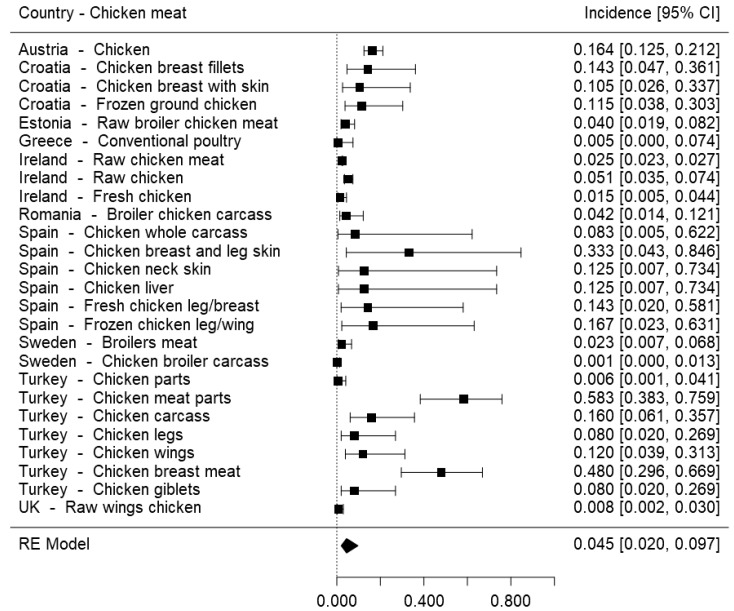
Forest plot of the incidence of *Salmonella* spp. in chicken meat surveyed in European establishments.

To gather the incidence measures retrieved from primary studies for *Campylobacter* spp. and *Salmonella* spp. in chicken meat, forest plots were built (Figure 2 and Figure 3). These pathogens were selected to assess their incidence per European country as they are both great concerns in terms of food safety, but one has currently very high incidence in this type of product, with more control still being needed, while the other is nowadays better controlled, after the implementation of various control processes specific to *Salmonella* spp.

Figure 2 reveals that the highest overall frequencies of *Campylobacter* spp. in chicken meat were reported in Italy (99.5%; 95% CI: 96.7–99.9%) and Northern Ireland (93.7%; 95% CI: 84.3–97.6%). Regarding *Salmonella* spp., Figure 3 shows that Turkey and Spain reported the highest overall frequencies of this pathogen in chicken meat, with prevalence values as high as 58.3% (95% CI: 38.3–75.9%) and 33.3% (95% CI: 4.30–84.6%), respectively.

Conclusions should be carefully taken from these plots, as the results might reflect more than just values of prevalence: for example, in Spain, coverage of the surveillance system for campylobacteriosis has improved and the number of reported confirmed cases has almost doubled since 2012 [91]. In any case, it can be stated that these forest plots consolidate the fact that *Salmonella* spp. is currently better controlled (from the incidence point of view but also the quality of the surveillance system) throughout the entire food chain in comparison to *Campylobacter* spp., as the prevalence of the latter is significantly higher than the first. It also emphasizes the pressing need of establishing control processes for *Campylobacter* spp. as effective as the ones in place for *Salmonella* spp., and that might not be the same in different countries, as previously stated by Skarp et al. (2015) [93].

## 4. Discussion

### 4.1. Incidence of S. aureus

The general meta-analysis, that is, with all data collected without any partitions, reveals that *S. aureus* is the pathogen of highest prevalence in overall poultry meats. According to Pepe et al. (2006) [94], staphylococci is one of the most common bacteria present in poultry slaughtering and processing environments, which is corroborated by the results of the sampling stage meta-analysis, in which *S. aureus* presented the highest prevalence in chicken meat surveyed at the end of processing (38.1%; 95% CI: 14.2–69.7%), but also at retail level (51.6%; 95% CI: 31.8–70.9%). The high prevalence at the end-processing stage is likely to be a consequence of the lack of control steps or the inefficiency to avoid contamination by this pathogen from the beginning of the process, at the slaughterhouses, where the skin and mucous membranes of animals are often contaminated, whether it is naturally or due to cross-contamination with infected carcasses. At retail level, there is not always a strict temperature control, making the products susceptible to this pathogen through various routes of contamination. As the vegetative form of *S. aureus* requires temperatures above those used for refrigeration to grow to levels of concentration of public health relevance [8], this is a relatively easy microorganism to control if the cold chain is secured throughout production, in comparison to *L. monocytogenes,* for instance, which grows even at refrigeration temperatures. In this sense, it is easier to reduce the health risks associated with this pathogen, which can explain the currently reduced effects on public health and low number of diseased.

Regarding the different cutting types, whole carcasses revealed to be the product with highest prevalence of pathogens, in particularly *S. aureus*, the one of greatest incidence. The trend observed among all analyzed microorganisms, that greater incidence occurs in whole carcasses in comparison with cut/minced meat and giblets, may be associated with the microbiological detection method used. In whole carcasses, microbiological analysis is preceded by homogenization of the entire carcass [54], meaning that the entire surface is sampled. On the contrary, analysis of pre-cut or minced meat and giblets are carried out by sampling 25 g of the product or swabbing 100 cm^2^ of the surface [75]. As it would be expected, sampling of whole carcasses is a much more sensitive technique in terms of pathogen detection in comparison to smaller samples of 25 g or 100 cm^2^ swabs, hence the higher prevalence detected.

For a mesophilic bacterium, *S. aureus* has a relatively high heat resistance [95], which can explain why some of the control steps implemented are not suitable for its inactivation. In particular, packaging was found insufficient to reduce the presence of this pathogen, as packed poultry meats revealed considerable prevalence of *S. aureus*, which may be caused by inefficient sterilization of the packaging material. Besides having a physical barrier to microorganisms, packed products are also subjected to a specific gas composition. Although the gas composition certainly impacts the spoilage of poultry meats, it is necessary to optimize the gas mixture that will affect bacterial growth the most [96].

Overall, special attention must be paid to *S. aureus* in undercooked meats and in chicken that will be used to produce transformed products such as sausages, as it is possible for *S. aureus* to survive and proliferate during further meat processing.

### 4.2. Incidence of Campylobacter spp.

The general meta-analysis showed that *Campylobacter* spp. is a pathogen of high prevalence in European poultry meats, which agrees with the statements of Humphrey et al. (2007), where poultry was pointed as an important source of campylobacteriosis, mostly because this bacterium is often carried in the intestinal tract of such animals [97].

*Campylobacter* spp. has a great colonization capacity, higher levels of intestinal carriage at slaughter than other bacterium (for instance *Salmonella* spp.) [98], and it is highly resistant to procedures such as scalding, washing and cooling, which generally reduce the incidence of other microorganisms to acceptable levels [97]. The reason for this survival may be the unique ability to attach to poultry tissues during carcass processing [98]. The incidence of *Campylobacter* spp. in the many chilled poultry meat samples surveyed across Europe confirms that, while cooling may be able to reduce the fast proliferation that generally occurs during the slaughter process, yet, it is insufficient to inactivate this pathogen [98,99]. The significantly lower prevalence estimated for frozen poultry (~10%), as opposed to the ~44% pooled prevalence in chilled poultry, was expected since freezing is known to decrease the number of campylobacters in time. In a fate study [100], it was found that *C. jejuni* inoculated in poultry meat samples decreased from 7.5 log CFU/g to 3.8 log CFU/g after only 30 min storage at −20 °C.

Regardless of the type of cut or packaging status, in chicken and other poultry meats, high incidence of this pathogen was observed. Furthermore, this meta-analysis also revealed high prevalence of *Campylobacter* spp. at the end of processing and retail level. Recently, studies are showing that campylobacters may be more robust than previously thought and represent a superior challenge to food safety [95], reason as to why the implemented safety control procedures during meat processing might be scarce or inadequate. With farms being the preliminary site of *Campylobacter* entry into production, the major intervention strategies should be targeted at farm level, enhancing biosecurity and implementing better monitoring, as interventions at slaughter process are less efficient [93].

There are plenty of opportunities for improvement along the food chain when it comes to the overall goal of reducing campylobacteriosis, with new and effective measures needing to be quickly implemented as *Campylobacter* has been the most commonly reported gastrointestinal bacterial pathogen in humans in the EU since 2005 [91]. In particular, *C. jejuni* and *C. coli* have been reported as the main causes of human campylobacteriosis [91,93].

### 4.3. Incidence of L. monocytogenes

Considered by EFSA as the second leading cause of food poisoning outbreaks [91], *Listeria monocytogenes* was found to be, in this meta-analysis, the third most incident pathogen in chicken and other poultry meats.

*L. monocytogenes* can be found naturally in the environment (soil, sewage, animal feces and water) [99,101] and in foods. In particular, poultry can be asymptomatic carriers of this pathogen and introduce contamination in slaughterhouses [102,103]. This is a great concern at the industrial level because this pathogen has the ability to withstand and adapt to various environmental stresses: it can multiply at low temperatures and form biofilms on food-processing equipment and food-contact surfaces, causing cross-contamination in chicken meat and its derivatives [102] and making a wide range of foodstuffs susceptible to contamination, which is particularly concerning for RTE foods that are not cooked or re-heated. The formation of these biofilms can be the cause for the high incidence values of *L. monocytogenes* found at the end-processing stage and retail level.

Commonly found in bird feces [104], *L. monocytogenes* high incidence in carcasses is likely to arise from cross-contamination during processing. At the end of the process, cooling of meat is intended to inhibit the multiplication of pathogens, but it was observed that in chilled poultry meats, the prevalence of *L. monocytogenes* was still quite relevant, confirming that refrigeration is not enough to inhibit the proliferation of this cold-resistant pathogen.

Following what was already observed for other pathogens, the occurrence of *L. monocytogenes* is higher in broiler meat commercialized without packaging than in packed ones. Packaging can stop further cross-contamination after the products are packed, but if the product is already contaminated with high levels of pathogens, if the wrapper is not properly sterilized, or if the inactivation processes used after packaging are not adequate, presence of pathogens can still occur.

### 4.4. Incidence of Salmonella spp.

In this work, we did not verify high levels of incidence of *Salmonella* spp. in poultry meat when compared to the other three pathogens under study. The highest prevalence found was in whole carcasses of poultry meat other than chicken, which is probably a consequence of the microbiological detection method, as previously stated.

It is evident that the low incidence of *Salmonella* spp. observed in this work is due to the high investments in zoonoses control in the past years. Since 2003, EU Member States have the responsibility to implement *Salmonella* national control programs and report the results to the European Commission and EFSA, as part of the annual EU zoonoses monitoring. These programs aim to reduce *Salmonella* prevalence in certain animal populations, particularly in breeding flocks of *Gallus gallus*, laying hens, broilers and breeding and fattening turkeys [91].

Currently, the establishment of strict biosecurity measures at farm level (including *Salmonella*-free poultry feed and water), vaccination programs in the parent flocks, and testing/removal of positive flocks from production have been used as control measures [104]. Additionally, the use of feed additives and acidified food and water have been encouraged, as the pH reduction is expected to have a protective effect on the feed, milling and feeding equipment and on the general environment [104].

This large reduction of salmonellosis and *Salmonella* prevalence in food products in the past years should be taken as a motivation for the control of other pathogens, as the effective implementation of control programs demonstrate that it is possible to produce safer food if the proper, adequate measures are implemented. However, it is crucial to keep in mind that the efficacy of such interventions depends on the level of bacterial contamination and that the control steps must be specific for each microorganism, as they have their own characteristics and different resistance to diverse conditions.

Despite the declining trend of salmonellosis in the EU that can give an increased sense of security, continuous research is mandatory to solve new difficulties, such as the increasing antimicrobial resistance in non-typhoid *Salmonella* species that has become a serious problem for public health worldwide [104]. Moreover, aiming for the reduction of one specific serovar or serotype is not sufficient, as the currently predominant serotypes in poultry flocks are likely to change over time [104].

## 5. Conclusions

It is a great concern that chicken meat has been considered one of the main causes of food poisoning while the poultry industry is simultaneously one of the most important sources of animal protein for the world’s population [102]. Pathogenic contamination of poultry can occur at any level: in the initial production environment, through vertical transmission (via egg, triggering the birth of infected chicks) or horizontal transmission (caused by contaminated environment or feed); or in the slaughtering process [6,47]. With gastrointestinal tract of birds and slaughtering facilities identified as the main reservoirs of poultry meat contaminants [96], it is necessary to implement preventive and corrective measures in several stages, but mainly on farm to reduce the initial levels of contamination that enter the process. As every stage of poultry meat production and processing systems has its own unique challenges regarding pathogen contamination and control, a multi-hurdle approach is likely to be the best strategy for pathogen reduction and elimination [105].

This meta-analysis on incidence data from European surveys indicated that *S. aureus* is currently the main contaminating pathogen of poultry meat, followed closely by *Campylobacter* spp. The establishment of control processes specific to these pathogens, as it was done in *Salmonella* spp. control programs, will certainly have a great impact on their current values of incidence, enabling the production and provision of safer meat products to consumers.

Adequate interventions at the processing stages can be assessed through challenge tests and predictive microbiology. In particular, growth and inactivation models can take into account factors such as the levels of contamination when carcasses leave the processing plant, storage time in retail stores, transport time, storage times in homes and the temperatures carcasses were exposed to during each of these periods [104], making this a unique tool for researchers and food companies to increase food safety and prevent new outbreaks.

Despite industries’ responsibility to take action on these matters, consumers should be further educated and encouraged to take preventive measures to ensure their health and well-being, such as: sanitization of hands, surfaces and utensils before and after handling poultry meat; separation of raw poultry meat from other foods (especially cooked) to avoid cross-contamination; proper storage of products, ensuring refrigeration temperatures under 5 °C; consumption of properly cooked, non-washed, poultry meat, as washing can spread bacteria and contaminate kitchen surfaces.

## Figures and Tables

**Table 1:** Meta-analysis of the incidence of pathogens in poultry meat by type of cut. Heterogeneity analysis comprises between-study variability to total variability ratio (*I*^2^) and proportion of between-study variability explained by type of meat cut (*R*^2^).

Microorganism	Product (*n*)	Pooled Prevalence	95% CI Pooled Prevalence	Heterogeneity
***Campylobacter* spp.**	**Chicken (30)**	0.486	(0.281–0.696)	*I*^2^ = 0.419 *R*^2^ = 0.251
	Pre-cut (17)	0.305	(0.115–0.595)	
	Whole carcass (11)	0.719	(0.432–0.896)	
	**Other Poultry (20)**	0.230	(0.090–0.472)	*I*^2^ = 0.211 *R*^2^ = 0.204
	Pre-cut (10)	0.248	(0.103–0.487)	
	Whole carcass (8)	0.311	(0.138–0.559)	
***L. monocytogenes***	**Chicken (45)**	0.210	(0.141–0.301)	*I*^2^ = 0.330 *R*^2^ = 0.046
	Pre-cut (33)	0.202	(0.125–0.311)	
	Whole carcass (9)	0.246	(0.131–0.414)	
	**Other Poultry (30)**	0.129	(0.078–0.204)	*I*^2^ = 0.408 *R*^2^ = 0.012
	Pre-cut (16)	0.117	(0.063–0.207)	
	Whole carcass (9)	0.143	(0.058–0.310)	
	Mince (5)	0.190	(0.073–0.409)	
***Salmonella* spp.**	**Chicken (26)**	0.032	(0.016–0.064)	*I*^2^ = 0.455 *R*^2^ = 0.154
	Pre-cut (18)	0.058	(0.027–0.122)	
	Whole carcass (5)	0.023	(0.007–0.068)	
	Giblets (3)	0.015	(0.003–0.059)	
	**Other Poultry (25)**	0.057	(0.027–0.119)	*I*^2^ = 0.285 *R*^2^ = 0.176
	Pre-cut (17)	0.058	(0.026–0.125)	
	Whole carcass (4)	0.141	(0.049–0.343)	
	Mince (4)	0.119	(0.032–0.357)	
***S. aureus***	**Chicken (16)**	0.399	(0.198–0.640)	*I*^2^ = 0.483 *R*^2^ = 0.246
	Pre-cut (7)	0.283	(0.090–0.612)	
	Whole carcass (3)	0.726	(0.386–0.918)	
	Giblets (6)	0.430	(0.169–0.736)	
	**Other Poultry (11)**	0.431	(0.204–0.692)	*I*^2^ = 0.313 *R*^2^ = 0.157
	Pre-cut (6)	0.298	(0.151–0.502)	
	Whole carcass (5)	0.413	(0.189–0.679)	

**Table 2:** Meta-analysis of the incidence of pathogens in poultry meat surveyed in Europe by sampling stage.

Pathogen	*n*	Pooled Prevalence	95% CI Pooled Prevalence
*Campylobacter* spp.			
End-processing	9	0.307	(0.118–0.594)
Retail	41	0.443	(0.281–0.618)
*L. monocytogenes*			
End-processing	14	0.217	(0.084–0.458)
Retail	61	0.171	(0.111–0.255)
*Salmonella* spp.			
End-processing	16	0.054	(0.016–0.161)
Retail	35	0.104	(0.053–0.193)
*S. aureus*			
End-processing	5	0.381	(0.142–0.697)
Retail	22	0.516	(0.318–0.709)

**Table 3:** Meta-analysis of the incidence of pathogens in chicken and other poultry meat by packaging status. Heterogeneity analysis comprises between-study variability to total variability ratio (*I*^2^) and proportion of between-study variability explained by packaging status (*R*^2^).

Microorganism	Product (*n*)	Pooled Prevalence	95% CI Pooled Prevalence	Heterogeneity
***Campylobacter* spp.**	**Chicken**			*I*^2^ = 0.419 *R*^2^ = 0.018
	Packed (20)	0.472	(0.194–0.769)	
	Unpacked (9)	0.471	(0.131–0.841)	
	**Other Poultry**			*I*^2^ = 0.211 *R*^2^ = NA *
	Packed (18)	0.311	(0.195–0.456)	
***L. monocytogenes***	**Chicken**			*I*^2^ = 0.330 *R*^2^ = 0.034
	Packed (37)	0.185	(0.114–0.287)	
	Unpacked (8)	0.307	(0.114–0.605)	
	**Other Poultry**			*I*^2^ = 0.408 *R*^2^ = 0.047
	Packed (18)	0.125	(0.074–0.201)	
	Unpacked (12)	0.148	(0.079–0.260)	
***Salmonella* spp.**	**Chicken**			*I*^2^ = 0.455 *R*^2^ = 0.007
	Packed (17)	0.031	(0.010–0.097)	
	Unpacked (9)	0.048	(0.021–0.108)	
	**Other Poultry**			*I*^2^ = 0.258 *R*^2^ = NA
	Packed (25)	0.079	(0.039–0.150)	
***S. aureus***	**Chicken**			*I*^2^ = 0.483 *R*^2^ = NA
	Packed (15)	0.408	(0.165–0.708)	
	**Other Poultry**			*I*^2^ = 0.313 *R*^2^ = 0.157
	Packed (6)	0.298	(0.152–0.502)	
	Unpacked (5)	0.413	(0.189–0.679)	

(*) Not applicable.

**Table 4:** Meta-analysis of the incidence of pathogens in poultry meat by cold preservation type.

Stage	Microorganism	*n*	Pooled Prevalence	95% CI Pooled Prevalence
Chilled	*Campylobacter* spp.	45	0.439	(0.242–0.657)
	*L. monocytogenes*	74	0.177	(0.126–0.243)
	*Salmonella* spp.	48	0.071	(0.041–0.121)
	*S. aureus*	26	0.469	(0.308–0.637)
Frozen	*Campylobacter* spp.	5	0.098	(0.032–0.263)
